# Ecoimmunity in Darwin's Finches: Invasive Parasites Trigger Acquired Immunity in the Medium Ground Finch (*Geospiza fortis*)

**DOI:** 10.1371/journal.pone.0008605

**Published:** 2010-01-06

**Authors:** Sarah K. Huber, Jeb P. Owen, Jennifer A. H. Koop, Marisa O. King, Peter R. Grant, B. Rosemary Grant, Dale H. Clayton

**Affiliations:** 1 Biology Department, University of Utah, Salt Lake City, Utah, United States of America; 2 Department of Entomology, Washington State University, Pullman, Washington, United States of America; 3 School of Biological Sciences, Washington State University, Pullman, Washington, United States of America; 4 Department of Ecology and Evolutionary Biology, Princeton University, Princeton, New Jersey, United States of America; BMSI-A*STAR, Singapore

## Abstract

**Background:**

Invasive parasites are a major threat to island populations of animals. Darwin's finches of the Galápagos Islands are under attack by introduced pox virus (*Poxvirus avium*) and nest flies (*Philornis downsi*). We developed assays for parasite-specific antibody responses in Darwin's finches (*Geospiza fortis*), to test for relationships between adaptive immune responses to novel parasites and spatial-temporal variation in the occurrence of parasite pressure among *G. fortis* populations.

**Methodology/Principal Findings:**

We developed enzyme-linked immunosorbent assays (ELISAs) for the presence of antibodies in the serum of Darwin's finches specific to pox virus or *Philornis* proteins. We compared antibody levels between bird populations with and without evidence of pox infection (visible lesions), and among birds sampled before nesting (prior to nest-fly exposure) versus during nesting (with fly exposure). Birds from the Pox-positive population had higher levels of pox-binding antibodies. *Philornis*-binding antibody levels were higher in birds sampled during nesting. Female birds, which occupy the nest, had higher *Philornis*-binding antibody levels than males. The study was limited by an inability to confirm pox exposure independent of obvious lesions. However, the lasting effects of pox infection (e.g., scarring and lost digits) were expected to be reliable indicators of prior pox infection.

**Conclusions/Significance:**

This is the first demonstration, to our knowledge, of parasite-specific antibody responses to multiple classes of parasites in a wild population of birds. Darwin's finches initiated acquired immune responses to novel parasites. Our study has vital implications for invasion biology and ecological immunology. The adaptive immune response of Darwin's finches may help combat the negative effects of parasitism. Alternatively, the physiological cost of mounting such a response could outweigh any benefits, accelerating population decline. Tests of the fitness implications of parasite-specific immune responses in Darwin's finches are urgently needed.

## Introduction

**Figure 1:**
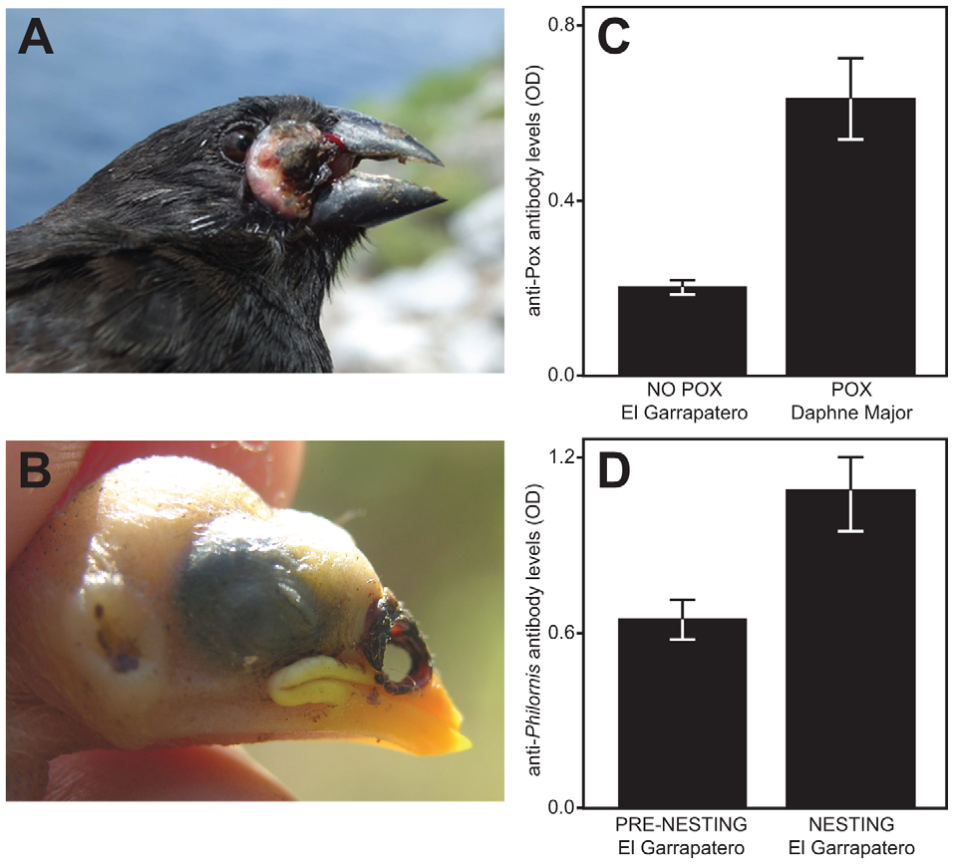
Parasite-specific antibody response of *Geospiza fortis*. (A) Medium ground finch, *Geospiza fortis*, with pox lesion in front of eye. (B) *G. fortis* nestling with *Philornis downsi* lesions in nostrils and ear. (C) Pox-binding antibody levels of adult birds on Daphne Major (n = 30) were higher than those of adult birds at El Garrapatero (n = 113) (Mann Whitney U = 619.50, p<0.0001). (D) *Philornis*-binding antibody levels of adult birds with active nests at El Garrapatero (n = 37) were higher than those of adult birds prior to nesting (n = 76) at the same site (U = 800, p<0.0001). Antibody response is measured as the optical density (OD) at 450nm. Bars indicate mean±standard error.

Invasive parasites pose a serious threat to native animal populations, because hosts with no history of exposure may lack effective immune defenses. Invasive parasites are a particular threat to small, island populations [1], [2]. For example, introduced malaria (*Plasmodium relictum*) has exacerbated the decline of Hawaiian honeycreeper species, many of which are now extinct [3], [4]. Darwin's finches have recently been exposed to two introduced parasites of high conservation priority: avian pox virus (*Poxvirus avium*) and the nest fly *Philornis downsi* (Figure 1A, 1B) [1], [2]. Both of these parasites have been shown to have negative effects on host fitness of Galápagos birds [5], [6], [7], [8], [9], [10]. If birds are able to mount an immune response to these novel pathogens, then they might ultimately be protected, to at least some degree, from the negative fitness consequences of parasitism. Alternatively, the physiological costs of an induced immune response to these parasites may exceed the benefits of mitigating parasite damage and contribute to negative fitness consequences. Indeed, these contrasting possibilities are a guiding force behind research within the field of ecological immunology [11].

The prevalence of *Avipox* in the Galápagos Islands varies on a geographic scale. Over the past 35 years it has been absent or very rare at Daphne Major and El Garrapatero, Santa Cruz Island. Daphne Major had episodic outbreaks of pox in 1983 and 2008 [12], and during our study in 2008, we found 50% of birds to be symptomatic for pox (15 out of 30 birds had active lesions). The outbreak of pox on Daphne Major in 2008 was not seen at El Garrapaterro. In 2008 not a single bird at El Garrapaterro, out of 129 individuals captured, was symptomatic, and none of these birds showed evidence of prior pox infection (e.g., scars or missing digits). The differences in pox prevalence between these two localities, allowed us to examine how infection influences pox-specific antibody levels in two populations with relatively similar histories of pox exposure.

*Philornis downsi* was first detected in the Galápagos in 1964; however, presence of the fly went relatively unnoticed until the late 1990's when large numbers of larvae were discovered in the nests of Galápagos land birds, including Darwin's finches [13], [14]. Adult flies are not parasitic, but larvae are obligate parasites that feed on the blood and tissues of nestling birds. Nestling Darwin's finches exposed to fly larvae have reduced survival and growth [8], [9]. At El Garrapatero in 2008, 96% of 23 nests were infested with *P. downsi*.

Ecological immunologists are exploring potential fitness trade-offs between immune defense against parasites and the physiological demands of other life-history traits (e.g. growth and reproduction). Although parasites are treated as a selective force acting on the immune system, few studies within ecological immunology use parasite-specific assays of immune function [15]. Non-specific assays do not clarify interactions between the immune system and parasites [16], [17]. As a result, non-specific assays do not directly test fitness effects of immunological variation in the context of parasite pressure. Here we take the first step in examining avian responses to introduced parasites directly, by demonstrating parasite-specific antibody responses to multiple classes of parasites in Darwin's finches. We developed assays for parasite-specific antibody responses in the medium ground finch (*Geospiza fortis*) (see Methods). Our goal was to test for relationships between adaptive immune responses to novel parasites and spatial-temporal variation in the occurrence of parasite pressure among *G. fortis* populations. Our results demonstrate that Darwin's finches produce antibodies against these invasive parasites, and that the immune responses are correlated with spatial-temporal variation in parasite pressure, both between finch populations, and between sexes. To our knowledge, this is the first time parasite-specific immune responses have been demonstrated relative to multiple classes of parasites in a wild population of birds.

## Results

Adult birds on Daphne Major had significantly higher levels of pox-binding antibodies than birds from El Garrapatero (mean±SE for Daphne Major = 0.63±0.09 optical density (OD); mean±SE for El Garrapatero = 0.20±0.02 OD; Mann Whitney U = 619.50; p<0.0001; Figure 1C).

When we compared *Philornis*-specific antibody levels in adult birds sampled before nesting (prior to *Philornis* exposure) with a different set of individuals sampled during the nesting period, we found significantly greater levels of *Philornis*-specific antibodies during the nesting period (mean±SE for nesting = 1.08±0.12 OD; mean±SE for pre-nesting = 0.64±0.07 OD; Mann Whitney U = 800.00; p<0.0001; Figure 1D).

We found no sex difference in pox-specific antibody levels (mean±SE for Daphne Major females = 0.61±0.12 OD; mean±SE for Daphne Major males = 0.67±0.18 OD; Mann Whitney U = 91.50, p = 0.71), suggesting equal exposure of males and females to pox virus.

In contrast, we found significantly higher *Philornis*-specific antibody levels in females compared to males (mean±SE for El Garrapatero females = 0.99±0.11; mean±SE for El Garrapatero males = 0.58±0.06; Mann Whitney U = 1018.00, p = 0.001). This result is consistent with adult females having increased exposure to *P. downsi* when they brood offspring (males do not brood).

## Discussion

Higher levels of pox-binding and *Philornis*-binding antibodies in Darwin's finches exposed to these parasites confirms that these birds are capable of mounting parasite-specific adaptive immune responses to novel parasites. Importantly, these antibody responses are directed against parasites that represent distinct immunological demands (intracellular versus external), and which constitute a serious threat to Darwin's finches. From the perspective of vertebrate immunology, it is not unusual that *G. fortis* is able to develop antibodies against novel challenges. However, our data are unique in two respects. This study is the first demonstration, to our knowledge, of ectoparasite-specific antibodies in a wild bird population. This study is also the first demonstration of parasite-specific antibodies directed against two distinct classes of parasites (external and intracellular) in a wild bird population. Within the field of ecological immunology, these observations are important because they establish a definitive immunological link between actual parasites and an animal of ecological interest [16].

These data also raise intriguing questions about prevailing assumptions regarding the host-parasite interactions of *P. downsi*. We found no differences in the levels of pox-binding antibodies between male and female finches. This finding agrees with the known ecology of avipox virus, which is transmitted by mosquitoes, or through bird-bird contact [1], [7], where no bias in transmission among the sexes would be expected. In contrast, we found significantly higher *Philornis*-specific antibody levels in females compared to males, which agrees with the expected bias of higher female exposure to *P. downsi* during female brooding on the nest. Thus, our data cast doubt on the assumption that adults are never bitten [18].

The prevailing notion that adults are not exposed to larval feeding is based primarily on two observations: (i) lesions from larval feeding have not been observed on captured adult females; and (ii) the scaly covering on the females legs is thought to prevent larvae from penetrating the female's skin. The absence of obvious lesions on females does not rule out the possibility that adult females are bitten. For example, fewer than half of the nestlings in our study had visible lesions associated with larvae feeding, even though nests were heavily parasitized and in many cases nestlings died (unpublished data). Second, while larvae likely could not penetrate the scales on female's legs, females might be vulnerable to larval feeding through their brood patch, which is completely devoid of a feather covering. Larvae may come into contact with the female's brood patch while she is sitting on nestlings, particularly when larvae are in the first or second instar and reside on the nestlings (e.g., in the nostrils or on the wing webbing) [18].

Although the immunological data indicate feeding attempts on females do occur, we are not suggesting this is evidence that adult finches are viable hosts for *P. downsi*. Blood feeding attempts on adult birds may consistently fail for a variety of physical and behavioral reasons. However, if feeding attempts by larvae are occurring, it is reasonable to expect adult females are exposed to *P. downsi* antigens that are stimulating an immune response. The ecological importance of this immune response depends on multiple unexplored factors. For example, antibody development by the female could confer a defensive advantage to offspring, if there is transfer of maternal antibodies to the chicks [19]. If females are exposed during the first clutch and produce antibodies, they might transfer these antibodies to the eggs of their second or third clutch. Alternatively, a stimulated antibody response in the female could produce a physiological demand that reduces energy available for foraging and subsequent breeding attempts in the season. A number of important immunological questions must be answered to address these possible ecological outcomes. For example, how quickly are antibodies produced and how long do they persist? Though anti-ectoparasite antibodies can be produced rapidly (1-week) and persist up to two months without stimulation [20], [21], the dynamics of anti-*Philornis* antibodies remain to be determined. We are currently attempting to determine if maternal antibodies are transferred to *G. fortis* offspring, as well as the timing of primary and secondary immune responses to *P. downsi* by female finches through the breeding season.

A critical next step in understanding the relationship between parasite infection and antibody production is to examine how these factors affect fitness. The only fitness data available for the effects of pox on Darwin's finches underscore the need for a detailed study of survival in relation to antibody response. Observations of *G. fortis* on Daphne Major in 2009 found that 11 out of 14 birds with pox symptoms in 2008 survived to the next year, compared with 12 out of 19 birds without pox symptoms (Fisher's exact test: two-tailed p = 0.46). These data suggest pox might not have the same impact on Darwin's finches as it does on Galápagos Mocking birds [5], [12], [22], [23]. However, long-term fitness effects estimated in relation to short-term measures of prevalence are inadequate for several reasons. First, we do not know the severity of pox infection for individuals in our study. We only know that some birds on Daphne Major were exposed, whereas birds at El Garrapatero were not exposed over the course of our study. Variation in the intensity of exposure is likely related to survival. Second, we do not know if birds that were unexposed to pox at the time of sampling continued to be parasite-free. Finally, survival may be confounded by sex, age, condition, and breeding status, among other variables. For example, males and females might have different physiological responses to these diseases or the costs of breeding might be greater in one sex than the other. For example, some evidence suggests that males with prior pox exposure might have decreased pairing success [7]. We emphasize the need for future studies that control for these factors and that experimentally test for the impact of parasite load and antibody production on fitness. For example, survival data for birds with controlled exposure to pox can be compared between individuals with low versus high levels of anti-pox antibodies; these data would allow us to test the extent to which antibody production might be protective. Conversely, survival data for birds that are known to be free of active pox infection can be compared between individuals with anti-pox antibodies and those without anti-pox antibodies; these data would allow us to test whether antibody production might be costly. Studies such as these should be a major focus of future research, for both pox and *Philornis*.

In summary, the assays presented here are valuable tools for exploring the ecological immunology of Darwin's finches, and in helping to determine the epidemiology of two critically important diseases threatening avifauna in the Galápagos archipelago. Broadly, we expect this approach can be applied to other research systems as well, which will strengthen studies that have typically relied on non-specific measures of immune function [16].

## Methods

### Ethics Statement

All procedures were approved by the University of Utah Institutional Animal Care and Use Committee (protocol #07-08004).

### Sample Collection

We studied birds at two sites in the Galápagos Islands: El Garrapaterro, Isla Santa Cruz, and Isla Daphne Major. Birds were sampled at El Garrapaterro from January–April 2008 and at Daphne Major on March 11, 2008. They were captured using mist nests, or Potter's traps, and each bird was individually marked with a combination of one aluminum ring and three darvic color bands. We noted whether birds had active pox lesions, or evidence of prior pox infection (e.g., missing digits). We then collected a small volume of blood by piercing the ulnar vein with a 27-gauge needle. Approximately 50 µl of blood was collected with a capillary tube and expelled into centrifuge tubes. Centrifuge tubes were stored on ice in the field (approximately 6 hours), then transported to the laboratory where they were centrifuged. The serum was then pipetted off the top and stored at −80°C.

At El Garrapatero, we made focal observations of individuals to determine pairing status and nest location. We checked nests every other day to determine egg laying date, clutch size, and hatch date. When nests were no longer active (nestlings were predated, fledged, or died), the nests were dissected to obtain fresh *Philornis downsi* larvae, which were placed in a centrifuge tube and stored at −80°C for future antigen extraction (see below).

Adults sampled at El Garrapatero were assigned to one of two groups: un-exposed or exposed. Un-exposed birds (n = 76) were individuals that 1) had a nest but were sampled prior to the hatching of their first brood, 2) females that did not have a brood patch (and thus were not breeding), or 3) unmated males that were sampled early in the breeding season. Exposed birds (n = 37) were those sampled while they had nestlings in the nest and had parasites present in the nest. No unexposed individuals were re-sampled during the nesting period, and no exposed individuals were sampled prior to the nesting period.

For birds sampled at Daphne Major and El Garrapatero the sex was determined based on plumage (black plumage for males and the presence of a brood patch for females) or by genotyping. Blood samples of individuals for which we could not determine sex (nonbreeding females and young males have identical plumage) were sent to Avian Biotech International (Tallahassee, FL) for genotyping via PCR. On Daphne Major we sampled 10 females and 20 males; at El Garrapatero we sampled 56 females and 57 males.

Comparisons of pox immune response were made between populations (Daphne Major versus El Garrapatero). We did not compare asymptomatic and symptomatic birds within populations because it was not possible to evaluate the timing of prior pox exposure from current symptoms alone. Asymptomatic individuals could have elevated antibody levels due to prior infection. Additionally, there is a lag between infection and the production of antibodies (10–12 days). Thus, symptomatic individuals could have low Pox-specific antibody levels due to sampling prior to antibody production. These factors confounded our ability to detect relevant differences in Pox-specific antibody levels within a population.

In contrast, we were able to compare *Philornis*-specific antibody levels between unexposed and exposed birds from El Garrapatero, because we could determine the timing of parasite exposure (nesting period), visually confirm the presence of the parasite, and obtain blood samples after the lag time required for up-regulation of any antibody response. Although pre-nesting birds could have been exposed to *Philornis* in a previous breeding season, and thus have anti-*Philornis* antibodies, we expected those antibody levels to be low (at or near background), owing to the breakdown of antibodies in the absence of antigenic stimulation between breeding seasons [24].

### Antigen Production

First and second instar larvae of *P. downsi* were used for antigen extraction. Larvae were placed into a centrifuge tube and macerated with 100 µL of phosphate buffered saline (PBS) and 1mM EDTA. The tube was centrifuged at 14.8 thousand revolutions per minute, and the supernatant containing the extract was removed. The supernatant was passed through a 0.2 micron filter and the protein concentration was estimated using a spectrophotometer. The extract was diluted to a concentration of 0.613 mg mL^−1^.

For pox antigen we used a live virus vaccine for Fowl Pox Virus (FP-VAC; Intervet/Schering-Plough), following tests of binding by Darwin's finch antibodies (see below) and based on the likely occurrence of conserved antigens among Fowl Pox and Canary Pox [25].

### Production of Secondary Antibody and Cross Reactivity with Darwin's Finch Serum

Anti-house-sparrow-immunoglobulin antiserum was produced by immunizing rats with purified house sparrow (*Passer domesticus*) IgY (Yolk Immunoglobulin).

House sparrow IgY was isolated using thiophilic interaction chromatography (described in 26). The recovered fraction was analyzed via sodium dodecyl sulfate polyacrylamide gel electrophoresis (SDS-PAGE) on 12% slab-gels and stained with Coomassie Blue R-250 to confirm the presence of house sparrow IgY.

Lyophilized house sparrow IgY was then re-dissolved in PBS at 1 µg/µl and emulsified with an equal volume of complete Freund's adjuvant (CFA). Three rats received a subcutaneous primary injection of house sparrow IgY with CFA (50 µg of protein/100 µl emulsion was used per injection). Rats received booster shots containing house sparrow IgY with incomplete Freund's adjuvant (IFA) at 4-week intervals two times. Rats were exsanguinated 4 weeks after the final booster shot.

**Figure 2:**
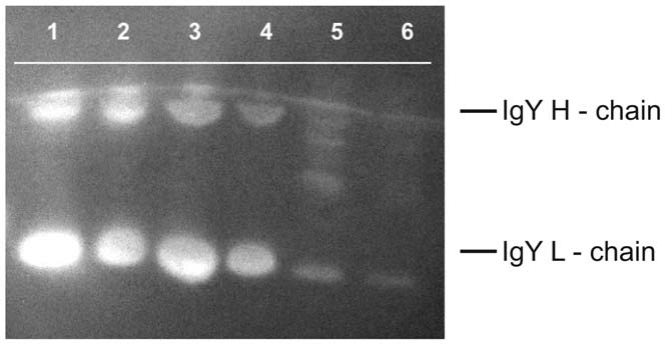
Western blot of serum dilutions developed for house sparrow IgY. Western blot of serum dilutions from Darwin's finch (DF), house sparrow and chicken using antibody markers developed for house sparrow IgY. Lane 1: DF serum 1∶10. Lane 2: DF serum 1∶20. Lane 3: house sparrow serum 1∶10. Lane 4: house sparrow serum 1∶20. Lane 5 chicken serum 1∶10. Lane 6 chicken serum 1∶20. Image indicates cross reactivity of house sparrow IgY detection antibody with Darwin's finch IgY. The lack of binding to chicken serum indicates no cross-reactivity with that species.

Cross-reactivity between house sparrow IgY, Darwin's finch serum and the rat antiserum was confirmed using Western-Blot analysis. Briefly, purified IgY was separated using SDS-PAGE and transferred on to a nitrocellulose membrane for immunoblotting. Filters were blocked with casein blocking buffer for one hour at room temperature and then washed three times in double deionized water (ddH2O). The blots were incubated for one hour at room temperature with rat-anti-house-sparrow-IgY (RαHOSP-IgY) and then washed three times again with ddH2O. The blots were then incubated for another hour at room temperature with commercially prepared goat-anti-mouse antibody conjugated to horseradish peroxidase (GαM-hrp) (Bethyl Laboratories, Inc., Mongomery, TX) and then washed a final three times with ddH2O. The blots were analyzed using enhanced chemiluminesence (Figure 2).

Cross-reactivity between Darwin's finch serum and RαHOSP-IgY was established via enzyme-linked immunosorbent assay (ELISA). Briefly, 96-well ELISA plates were coated in triplicate with 100 µl of Darwin's finch serum diluted at 1∶100, 1∶500, 1∶1000, and 1∶5000 in carbonate coating buffer (0.05 M, pH 9.6). The plates were incubated for one hour at 37°C on an orbital table before being washed three times with 200 µl of wash solution per well. The plates were blocked with casein blocking buffer and again incubated for one hour at 37°C on an orbital table. The RαHOSP-IgY was diluted in sample buffer at 1∶50, 1∶100, 1∶500 and 1∶1000. After washing the plate three times, 100 µl of the RαHOSP-IgY was added to each Darwin's finch serum dilution, such that each serum dilution was tested against each RαHOSP-IgY dilution. Plates were again incubated for one hour at 37°C on an orbital table and then washed three times. The secondary antibody, GαM-hrp, was diluted 1∶1000 in sample buffer and 100 µl of this solution was added to each well. The plates were incubated for one hour at 37°C on an orbital table and then washed a final three times. 100 µl of peroxidase substrate (2,2′-azino-bis-3-ethylbenzthiazoline-6-sulphonic acid, ABTS: Sigma cat. A1888) and peroxide was added to each well and the plates were covered with tinfoil and allowed to develop for one hour at room temperature before being read on a spectrophotometer using a 405-nanometer filter. Three blank wells were included on each plate, as well as three wells that measured non-specific binding, which quantified binding of RαHOSPIgY and GαM-hrp to the respective antigen. These wells received all the reagents described above except for Darwin's finch serum. In this step, blocking buffer was used in place of serum. The mean absorbance of these wells was subtracted from the absorbance measures determined above. Results from this ELISA indicated crossreactivity between Darwin's finch serum and RαHOSP-IgY.

### Cross Reactivity of Darwin's Finch Antibiodies and Parasite Antigen

**Figure 3:**
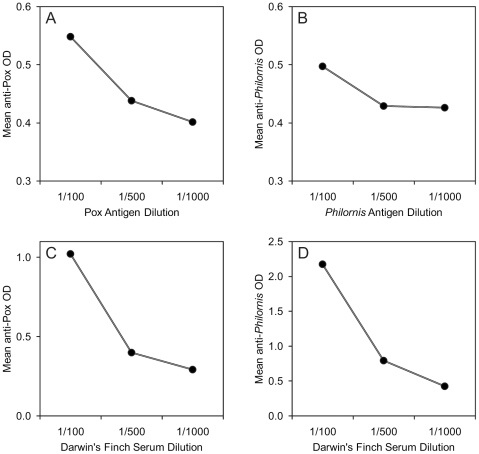
Optimization of ELISAs for antigen and Darwin's finch serum. Optical density (OD) values for optimization ELISAs of (A) Pox antigen dilutions and Darwin's finch serum at 1/500, (B) *Philornis* antigen dilutions and Darwin's finch serum at 1/500, (C) Darwin's finch serum dilutions and Pox antigen at 1/1000, and (D) Darwin's finch serum dilutions and *Philornis* antigen at 1/1000. Decreasing amounts of antigen (A,B) and antibody (C,D) result in decreasing optical density values, indicating specific antibody-antigen binding.

Cross-reactivity between Darwin's finch antibodies and *Philornis downsi* protein, or Fowl Pox virus, was established via ELISA, using dilutions of Darwin's finch serum and antigen (*Philornis* protein or Fowl Pox virus). Briefly, 96-well ELISA plates were coated in triplicate with 100 µl of either Fowl Pox virus in PBS, or *Philornis* extract, diluted at 1∶100, 1∶500, or 1∶1000 in carbonate coating buffer (0.05 M, pH 9.6). Plates were incubated for one hour at room temperature on an orbital table, and then washed five times in wash buffer. Wells were then coated with 200 µl bovine serum albumin (BSA) blocking buffer, incubated for 30 minutes at room temperature on an orbital table, and then washed five times with wash buffer. Each well was then loaded with 100 µl of Darwin's finch serum (pooled sample) then diluted 1∶100, 1∶500 or 1∶1000 in sample buffer, such that each serum dilution was tested against each antigen dilution. Plates were incubated for one hour at room temperature on an orbital table, and then washed (5×) with wash buffer. Next, 100 µl of RαHOSP-IgY (1∶1000) was added to each well, followed by a one hour incubation at room temperature and wash (5×). The second detection antibody (GαM-hrp, 1∶1000) was then added, followed by a one hour incubation at room temperature and washing (5×). Finally, 100 µl of peroxidase substrate (tetramethylbenzidine, TMB: Kirkegaard and Perry cat. 50-77-03) was added to each well. The plates were incubated for exactly five minutes at room temperature and the reaction was stopped using 100 µl of 2 M H_2_SO_4_ in each well, before reading optical density on a spectrophotometer using a 450-nanometer filter. Based on optimization results (Figure 3), a standard serum dilution of 1∶500 was selected for the ELISAs of individual birds and a standard dilution of 1∶1000 was selected for Pox and *Philornis* antigens, which were tested separately.

On each plate we included three wells for non-specific binding, which quantified binding of RαHOSP-IgY and GαM-hrp to the respective antigen. These wells received all the reagents described above except for Darwin's finch serum. In this step, blocking buffer was used in place of serum. The mean absorbance of these wells was subtracted from the absorbance measures determined above. Finally, we calibrated absorbance values between plates using a positive control. In brief, each plate contained the same reference sample in triplicate. The reference sample absorbance was compared across all plates, and we calculated a correction factor for each plate to standardize absorbance. These standardized values were used for subsequent analyses of immune response in birds.
